# Management of Hydrofluoric Acid Burns in the Emergency Department

**DOI:** 10.7759/cureus.7152

**Published:** 2020-03-01

**Authors:** Jason Lippert, Bobby Desai, Michael Falgiani, Trilok Stead, Latha Ganti

**Affiliations:** 1 Emergency Medicine, Ocala Regional Medical Center, University of Central Florida College of Medicine, Ocala, USA; 2 Emergency Medicine, Trinity Preparatory School, Winter Park, USA; 3 Emergency Medicine, Envision Physician Services, Orlando, USA; 4 Emergency Medicine, University of Central Florida College of Medicine/Hospital Corporation of America Graduate Medical Education Consortium of Greater Orlando, Orlando, USA; 5 Emergency Medicine, Polk County Fire Rescue, Bartow, USA

**Keywords:** hydrofluoric acid, calcium gluconate

## Abstract

Hydrofluoric acid burns are uncommon but unique among chemical burns in that they can cause visually mild burns with significant deep tissue injury and systemic toxicity through multiple mechanisms. We present the case of a patient who presented with bilateral hydrofluoric acid burns to his hands from aluminum brightener. The patient had been using an aluminum brightener with a hydrofluoric acid concentration of 10% for several months at work. On emergency department presentation, the patient endured significant tenderness to his hands and fingers. The patient suffered no serious complications, had no concerning lab or electrocardiographic findings, and was treated symptomatically with calcium gluconate gel. He was discharged home after successful symptom resolution with proper return precautions and instructions on how to safely use hydrofluoric acid containing products. Although not a very common cause of burns, acute care of these burns requires specific knowledge which is imperative for emergency personnel.

## Introduction

Hydrofluoric acid (HFA) is an inorganic acid commonly used in both domestic and industrial settings for cleaning agents, fertilizer production, and many other uses [1,2]. In the household, HFA can be found in products such as silverware cleaner and herbicides. In the laboratory, HFA is used to remove rust, etch glass, and clean brass and crystal. It is also used in the production of semiconductor chips and gasoline [3]. In the dental industry, HFA is used as an etchant agent for ceramic materials [2].

Although a weak acid, at concentrations > 20%, HFA can cause life-threatening caustic injury. In addition to corrosive burns, HFA is lipophilic, leading to deeper burns with liquefaction necrosis. On a systemic level, HFA releases fluoride ions that bind to calcium and magnesium, leading to hypocalcemia and hypomagnesemia, respectively. As a result, HFA can lead to significant systemic effects such as hypotension, cardiac arrhythmias, and cardiac failure [1].

Classically, physical examination demonstrates pain out of proportion to exam. Patients may have a delayed onset of symptoms of up to 24 hours, depending on the concentration. Patients can develop necrosis, ulceration, or gray areas at sites of exposure.

## Case presentation

A 37-year-old male presented to our emergency department (ED) with bilateral, burning hand pain, specifically under his nail beds and at his fingertips. The patient was exposed to aluminum brightener while at work, involving both of his hands. The patient did not use gloves and had been working without any issues for several months before this presentation. The product had an HFA concentration of 10%.

The patient was hemodynamically stable on presentation, including initial vitals of heart rate of 86 beats per minute, respiratory rate of 18 breaths per minute, and a blood pressure of 138/78 mmHg. On physical examination, the patient had bilateral tenderness to both hands, but did not demonstrate any external findings such as erythema, necrosis, ulcerations, or discoloration. No significant neurological or vascular deficiencies were found for either extremity. The remainder of the physical examination was unremarkable.

The patient’s medical workup included an ECG, basic metabolic panel, complete blood count, and magnesium and calcium concentrations. The local poison control center was contacted, who agreed with the workup and recommended applying calcium gluconate (CaG) gel to the affected areas until the patient had complete relief of his pain. 

ECG was negative for significant findings, including interval prolongations and arrhythmias. All lab results were within normal limits, including calcium and magnesium concentrations. The patient was treated with CaG gel, made in the ED by taking one gram of CaG and mixing it with standard lubricating gel in the ED. He received a total of three treatments of this gel before he had complete resolution of his pain.

Per the poison control center, once the patient was asymptomatic, with a normal ECG and lab values, he could be discharged home. The patient was given instructions concerning return precautions and instructions on how to safely use HFA products, including the use of appropriate gloves. He was then discharged home.

## Discussion

HFA burns are uncommon but unique among chemical burns in that they can cause visually mild burns with significant deep tissue injury and systemic toxicity through a variety of mechanisms. The majority of medical literature discussing these burns is derived from case reports, small case series, animal studies, and anecdotal evidence [1].

HFA has multiple mechanisms of injury (Table 1), leading to the wide range of presentations. At high enough concentrations, HFA releases hydrogen ions, leading to visible tissue destruction, similar to other acid burns [1,4]. However, the majority of HFA burns are low concentration, and do not present with immediate corrosive destruction, and also may not have immediate pain [1]. At lower concentrations, HFA is lipophilic, leading to deep tissue injury due to to liquefaction necrosis. Once the fluoride ions dissociate they are easily absorbed into the bloodstream. These ions may then bind to calcium and magnesium cations, leading to decreased serum concentrations and their associated systemic effects [1,2]. Hypocalcemia is associated with perioral numbness, paresthesias, muscle cramps, focal or generalized weakness, and peripheral neuromuscular irritability that manifests as tetany. Depletion of calcium causes inhibition of the sodium potassium ATPase pump which results in cellular membrane permeability of potassium leading to hyperkalemia [5].

**Table 1:** Mechanisms of action of hydrofluoric acid (HFA)

Mechanism	Effects
Caustic injury	Hydrogen ion release results in visible tissue destruction, ulceration, and necrosis upon contact. Most pronounced at higher HFA concentrations.
Liquefaction necrosis	Fluoride ion travels to deeper layers of skin, destroying nerves, blood vessels, and soft tissue.
Chelation	Fluoride ion binds to calcium and magnesium, resulting in hypocalcemia and hypomagnesemia, respectively. Together these lead to hyperkalemia. Electrolyte imbalances lead to cardiac dysrhythmias.

Hypomagnesemia is associated with neuromuscular excitability and cardiac dysrhythmias. ECG abnormalities that can occur include prolonged QTc (from hypocalcemia), QRS widening (from hyperkalemia), polymorphic ventricular tachycardia (from hypomagnesemia), and T-wave elevation [5]. These cardiac dysrhythmias put HFA-exposed patients at risk for sudden cardiac death [2].

Skin symptoms are directly related to the acid concentration. Concentrations greater than 50% can cause immediate severe throbbing pain, with whitish discoloration of the skin and vesicles surrounded by an erythematous flare.These eventually transform into blisters containing necrotic tissue [2]. Should the burn affect the hands or fingers, reduced motor activity, decreased sensitivity, and even ischemia may result from arterial vasospasm [2]. More dilute solutions (concentrations ranging from 20% to 50%) cause significant injury as well; however, symptoms may be delayed up to eight hours [4]. Lower concentrations (below 20% HFA) produce symptoms that tend to be delayed from 12 hours to up to several days after exposure, with no immediate pain [2]. The characteristic “pain out of proportion to exam” that these burns are known for is due to local neural hypocalcemia causing neuronal misfiring resulting in neuropathic pain.

Systemic toxicity is associated with any burns >50% concentration, and exposure of >5% of total body surface area regardless of concentration [6,7]. In addition to the electrolyte imbalances and cardiac dysrhythmias noted above, an excess of fluoride ions may lead to renal dysfunction, insufficiency, and renal cortical necrosis [2].

**Figure 1:**
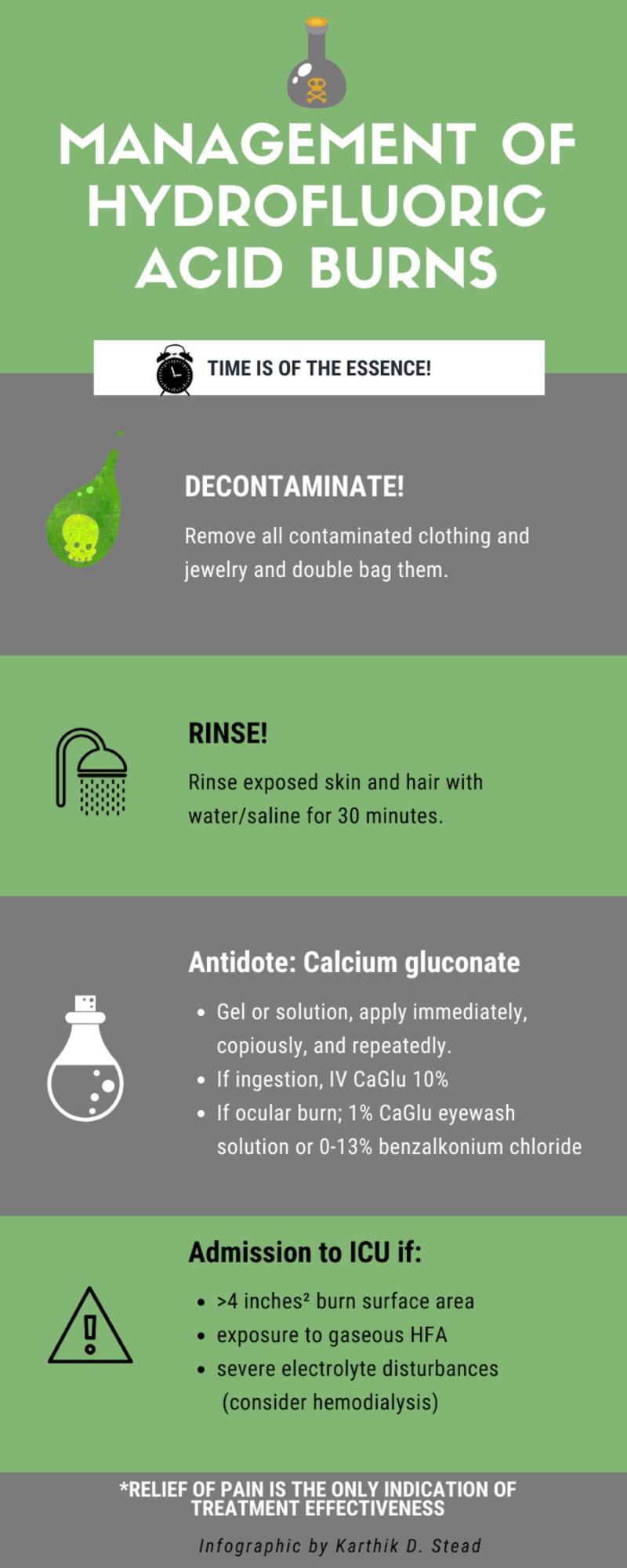
Management of hydrofluoric acid burns

The initial treatment of HFA burns starts with a quick airway-breathing-circulation assessment, removal of contaminated clothing, and double bagging these contaminated articles to prevent secondary exposure (Figure 1).

The goals of treatment are as follows: (1) skin or surface decontamination; (2) neutralization of the fluoride ion; and (3) minimizing ongoing HFA absorption. Decontamination is accomplished via irrigation with copious amounts of water, saline, or soap solution (pH 8 or above). Ice can be applied to the to affected part to produce vasoconstriction and slowing of HFA transport into the bloodstream [8].

Neutralizing agents include CaG, benzalkonium chloride, polyethylene glycol, magnesium oxide, or Hexafluorine® (Prevor, Nesles-la-Vallée, France) [9]. CaG is the primary neutralizing agent used and comes in a variety of formulations (solution, gel, or ointment) [2,8]. A 10% CaG solution or 0.13% benzalkonium chloride can be used for immersion of body parts (excluding the face) or for soaking compresses. In gel form, 2.5% CaG gel is rubbed into affected area for 15-30 minutes using HFA-resistant gloves. Calcium is used to bind to free fluoride ions, forming insoluble salts. Once CaF salt forms, the gel turns white. It should be wiped off and re-applied multiple times to mitigate the damage from fluoride ions [2]. Hexafluorine is a proprietary solution developed specifically for HFA burns. It simultaneously absorbs acid ions (H+) and fluoride ions (F-). Its chelation power is approximately 100 times that of CaG [9].

Pain is an important finding - lack of pain is the endpoint for treatment. As such, analgesic agents should be used with caution [7]. Generally acetaminophen and non-steroidal analgesics are reasonable. Throughout treatment, the patient’s electrolytes need to be monitored. The healthcare team needs to monitor the patient’s electrolytes, and correct hyperkalemia, hypocalcemia, and hypomagnesemia as clinically indicated. 

## Conclusions

HFA burns are the not most common form of burn injury seen in the ED; however, their unique properties can result in multiple forms of clinical injury. Our patient fortunately did not experience any systemic toxicity secondary to his chronic exposure. However, it is imperative that emergency personnel are aware of the acute management to optimize outcomes. 
